# Navigating Law Enforcement Presence in Emergency Departments

**DOI:** 10.1001/jamanetworkopen.2025.51804

**Published:** 2026-01-13

**Authors:** Prashasti Bhatnagar, Cassandra Ramdath, Desiree Pinto, Erin Hall

**Affiliations:** 1 Department of Sociology, UCLA (University of California, Los Angeles), Los Angeles; 2 Department of Surgery, MedStar Washington Hospital Center, Washington, DC; 3 Center for Innovations in Community Safety, Georgetown University, Washington, DC

## Abstract

**Importance:**

Law enforcement presence is widespread in emergency departments (EDs), but unregulated law enforcement exposure can be associated with poor health outcomes for survivors of violence (SOVs). Current literature has not explored the effective management of law enforcement presence in emergency medical settings that would support SOVs while allowing the priorities of other ED stakeholders (clinicians, staff, and law enforcement) to be met.

**Objective:**

To identify mutually beneficial strategies to manage and mitigate the unintended harms associated with law enforcement presence in the ED.

**Design, Setting, and Participants:**

This qualitative study was conducted between September 2020 and September 2023 in an urban level I trauma center. Trained researchers conducted semistructured interviews with hospital-based violence intervention program (HVIP) representatives and SOVs as well as focus groups with law enforcement officers (LEOs). Eligible participants included SOVs (survivors and affected family members), HVIP representatives (staff and clinicians) who had interacted with law enforcement in the ED, and LEOs (patrol officers, detectives, and captains) who had directly interacted with SOVs in the ED. Data analysis was conducted between July 2022 and October 2023.

**Main Outcomes and Measures:**

Multistakeholder perspectives on their experiences, needs, and priorities in the ED and recommendations for potential effective strategies to manage law enforcement in the ED. Mixed interviews and focus groups were used to tailor methods to the stakeholder groups and triangulate across stakeholders. A qualitative thematic analysis was used to synthesize data and develop themes. Transcripts were coded using codebooks, which were developed using study protocols and iteratively revised after rounds of independent coding.

**Results:**

In this study, 21 SOVs and 23 HVIP representatives were interviewed, and 16 LEOs participated in focus groups. Stakeholder consensus emerged on 3 main themes: triaging interactions with law enforcement, formalizing hospital staff and law enforcement training, and integrating SOV advocates in the ED.

**Conclusions and Relevance:**

This qualitative study identified 3 strategies, informed by the perspectives of multiple stakeholders, to manage law enforcement presence in the ED. Optimization of hospital environments through these 3 strategies may support SOV rights and recovery, mitigate adverse interactions between law enforcement and SOVs, and improve information sharing and job effectiveness for different stakeholders in the ED.

## Key Points

**Question:**

What are strategies to manage law enforcement presence in emergency departments (EDs)?

**Findings:**

In this qualitative study involving 21 survivors of violence (SOVs), 23 representatives of a hospital-based violence intervention program, and 16 law enforcement officers, 3 themes for managing law enforcement presence in the ED emerged. These included triaging interactions with law enforcement, formalizing hospital staff and law enforcement training, and integrating SOV advocates in the ED.

**Meaning:**

Findings of this study suggest that optimizing hospital environments by adopting strategies informed by multiple stakeholders can support SOV rights and recovery, mitigate adverse interactions between law enforcement and SOVs, and improve information sharing and job efficiency for different stakeholders in the ED.

## Teaser

This qualitative study examines the perspectives of survivors of violence, hospital staff and clinicians, and law enforcement officers regarding managing law enforcement in emergency medical settings.

## Introduction

Law enforcement presence in the emergency department (ED) is widespread, especially in urban, safety net hospitals.^1,2,3,4,5^ While in the ED, law enforcement officers (LEOs) frequently interact with hospital-based violence intervention program (HVIP) representatives and survivors of violence (SOVs), including patients and affected family members.^1,2^ HVIPs are a burgeoning intervention most commonly available at urban level I trauma centers.^6,7,8,9,10,11^ Although there is heterogeneity across HVIP models, the hallmarks include direct engagement with SOVs in the hospital by staff members who are credible messengers and provide trauma-informed support services.^6,7,8,9,10^ In this way, HVIP representatives often bridge the gap between SOVs and traditional hospital treatment teams through a combination of lived experience, trust building, and integration into medical teams.^6,7,8,9,10^ While SOVs, HVIPs, and LEOs have a vested interest in decreasing violence, there are ongoing reports of tension and unintended harms associated with law enforcement interactions in the ED. This study engaged voices from each stakeholder group and asked those most affected for potential policy changes to optimize interactions in the ED.

Despite frequent law enforcement presence in the ED, less than one-third of clinicians are aware of policies surrounding law enforcement presence, and those who are aware struggle to recite or clarify the policies.^12,13,14,15^ Informal or lack of hospital policies on law enforcement presence in the ED creates uncertainty among clinicians and patients and can have unintended consequences for patients, including interrupted medical care, compromised privacy and confidentiality, distrust in the patient-physician relationship, racial bias and disparities, and repeated trauma.^3,4,12,14,16,17^ It is important to note that the vast majority of urban gun violence is experienced by Black people.^11,18,19^ Thus, the tensions and potential harms of unregulated law enforcement activity in EDs may be particularly acute for the Black patient population.^11,18,19^

The lack of guidance from policies can place health care practitioners between the investigative priorities of law enforcement and patient care responsibilities, creating tension and moral distress for clinicians.^2,12,15,20^ Accordingly, formal hospital policies guiding law enforcement presence and training on effective implementation offer critical opportunities to protect SOVs; alleviate moral distress among clinicians; and provide better patient-centered, trauma-informed care.^2,12,15,20^ Given the paucity of literature, this study aimed to identify mutually beneficial strategies to manage and mitigate the unintended harms associated with law enforcement presence in the ED. The stakeholders in this study included groups most directly involved in the ED: SOVs, HVIP representatives, and LEOs. We defined SOVs broadly to include both patients who experienced direct harm and affected family members who experienced secondary trauma, loss, or disruption as a result.^21^

## Methods

This qualitative study was conducted between September 2020 and September 2023 at an urban level I trauma center with a high volume of SOVs and an active HVIP. The Georgetown-MedStar Research Institute Institutional Review Board deemed the SOV interviews and law enforcement focus groups exempt from ethics review, because they were determined to be part of a quality improvement project, and approved the interviews with HVIP representatives. Verbal informed consent was obtained from SOVs and LEOs before interviews and focus groups; HVIP representatives provided written consent via email before interviews. We followed the Consolidated Criteria for Reporting Qualitative Research (COREQ) reporting guideline.^22^

### Data Collection

Trained law students and medical students with experience in interviewing conducted semistructured interviews with HVIP representatives and SOVs. An experienced mixed-methods PhD-trained researcher (C.R.) conducted focus groups with law enforcement. Data collection occurred from September 2020 to September 2023 and concluded when saturation was reached, which was ascertained by ongoing review of transcripts by experienced researchers (P.B., C.R., and E.H.) until no new themes were emerging across successive interviews and focus groups. Among participating SOVs, patients received a $50 grocery gift card and affected family members received a $75 honorarium. Law enforcement focus groups were conducted during paid work time.

Interviews and focus groups aimed to elicit diverse stakeholder experiences and identify their needs and priorities in the ED, understand their knowledge of hospital policies on law enforcement, and generate policy and training recommendations. We conducted interviews with HVIP representatives and SOVs to allow for in-depth exploration of sensitive and emotionally charged experiences with care and privacy. Focus groups with law enforcement allowed us to capture collective perspectives and dialogue within the law enforcement culture as well as helped address the schedule constraints of officers. To support trust and minimize barriers to participation in this qualitative study, we made collection of demographic data optional. By mixing the interviews and focus groups, we were able to tailor methods to particular needs and contexts of the stakeholder groups and triangulate across stakeholders, strengthening the validity of our data.

The interview and focus group protocols were developed with insights from our study team, the HVIP staff, and law enforcement. Our team comprised a lawyer and qualitative researcher (P.B.); a mixed-methods researcher (C.R.); a surgical resident (D.P.); and a medical director of an HVIP, trauma surgeon, and mixed-methods researcher (E.H.), all of whom with experience in violence prevention, SOV and trauma care, and public health. Interview protocols for SOVs and HVIP representatives were pilot tested with local HVIP staff, who are survivors of community violence themselves and work closely with SOVs in the ED. Law enforcement focus group protocols were developed with input from a LEO and pilot tested with detectives, sergeants, and patrol officers.

SOV interviews were conducted in English or Spanish by native speakers in person and over the telephone, and HVIP interviews were conducted virtually. All interviews ranged from 30 to 60 minutes. In-person focus groups with law enforcement ranged from 60 to 120 minutes and included a mix of patrol officers, detectives, and captains.

### Data Analysis

Interviews and focus groups were audio recorded, transcribed verbatim (Otter.ai; Otter.ai Inc), and analyzed (Dedoose; Sociocultural Research Consultants LLC). Spanish interviews were transcribed (Speechmatics software; Speechmatics) and translated to English by a native Spanish speaker before coding. Data analysis was conducted between July 2022 and October 2023.

We used qualitative thematic analysis to synthesize data and develop themes.^23,24,25^ Codebooks were first developed deductively from the study protocols by an experienced qualitative researcher (P.B.). In an iterative process, our coding team tested for intercoder reliability by independently coding at least 3 different transcripts for each stakeholder group, meeting to resolve discrepancies, revising and finalizing the codebook, and identifying prevalent themes. The experienced qualitative researcher (P.B.) coded all transcripts. A trained undergraduate student coded law enforcement focus groups and HVIP interviews, and a trained medical student coded SOV interviews. Final codebooks consisted of mostly the same codes across groups to facilitate consensus building and coding reliability, with some variation in codes based on emergent data.

Analytic memos were drafted by the experienced qualitative researcher (P.B.) to compare coding, identify consensus, reduce bias, and facilitate triangulation between and across stakeholder groups. Analytic memos were discussed at our regular team meetings (P.B., C.R., D.P., and E.H.) to practice reflexivity, reach consensus on interpretations, and engage in data checking. Involvement of multiple team members in data collection, independent coding, and collaborative discussion enhanced the credibility and rigor of our findings by minimizing individual bias and promoting a more comprehensive interpretation of the data.

## Results

This analysis included perspectives from 60 individuals. Of these, 23 HVIP representatives and 21 SOVs participated in semistructured interviews. Three focus groups with law enforcement were conducted and involved 16 LEOs. We identified 3 main themes that stakeholder groups independently reached consensus on: (1) triaging interactions with law enforcement (Table 1), (2) formalizing hospital staff and law enforcement training (Table 2), and (3) integrating SOV advocates in the ED (Table 3).

**Table 1.** Quotations for Theme 1: Triaging Interactions With Law Enforcement

Theme	Quotations from stakeholders
Adverse outcomes of SOV and law enforcement interactions	“[Police] shouldn’t be allowed to come in until somebody is physically and mentally stable.... Don’t let [the] police harass patients, you know, while they are confused.” (SOV)
	“The policy of the hospital should be that the police officers can’t come and bully the nurse or go and question a patient if they are not coherent. It just should be just a law. I mean, if you got a HIPAA right or a law, then just add an amendment to it and just say, ‘You can’t question a patient if the patient is not coherent or if the patient is on some type of highly [sic] drugs or medication.’ So it should be some type of where you draw the line because the doctor’s not going to let you see a patient if they can’t talk or if they are on life support.” (SOV)
	“Police come to the bedside, the patient is drugged up [and] is hurting in pain. They are not in the right frame of mind...they can’t provide or be able to share the necessary information in the most accurate way. Because the cognitive bandwidth is just not there.” (HVIP representative)
Law enforcement’s need for timely patient condition updates	“Maybe have a form that [the hospital] could just fill out for us whenever a [survivor] of a crime comes in and we could just give a list of questions that we need to know right away…they can have it filled out…if we have follow-ups, we can ask it.” (Detective)
	“You can’t really leave the emergency room [without completing investigative priorities]. You can’t get any information over the phone [because of HIPAA]. If there was a way we could find the workaround for [getting updates on patient condition], that would be pretty helpful.” (Detective)
HVIP role with law enforcement	“We are not doing anything to hinder their [police] investigations.... Our goal is aligned with their goal, we are just going in a different way.” (HVIP representative)
	“We don’t want to impede their [police] investigation, but at the same time, we don’t want them violating the patient’s rights.” (HVIP representative)
	“Part of our job and the [HVIP] program...is to try to reduce emotional distress in the family. Because that’s often when retaliation is discussed, when emotions are high. And so if we can provide continuous medical updates, at least the family has information to cope.” (HVIP representative)

Abbreviations: HIPAA, Health Insurance Portability and Accountability Act; HVIP, hospital-based violence intervention program; SOV, survivor of violence.

**Table 2.** Quotations for Theme 2: Formalizing Hospital Staff and Law Enforcement Training

Theme	Quotations from stakeholders
Training on roles and rights for hospital staff	“When we talk about things like feeling empowered to tell the police ‘no,’ I say that with confidence from the physician faculty. I have no idea if a nurse would feel as comfortable saying something.… People [who] have less power in the system, they might feel more sensitive or manipulated.” (HVIP representative)
	“When a police officer or detective comes in with a [survivor] of violence and wants to meet with them and wants to take their belongings, people let them in.… People don’t feel like ‘Oh, I can just say no, you can’t come back here. Or no, you can’t take those things’.… We are trying to figure out where is our right as the hospital and what we are responsible to do for patients’ belongings versus what the police rights are.… I think I have heard from nursing staff, they don’t feel empowered to tell somebody [law enforcement] ‘no.’ And also, you know, they [nursing staff] are in an emergency. They are really focused on saving someone’s life.” (HVIP representative)
	“There is no training. There is no point in the academy where you’re role playing, ‘This is how you conduct yourself in the hospital with this kind of scene’.… You go approach this shot-up kid, who’s probably not in the mood to talk.… There’s no training for it.” (Patrol officer)
Trauma-informed training for law enforcement	“[Police] have an oath and they have a job, but I don’t think their job is dealing with the public, especially when our children die or our family members die. I just think that they need to have somebody with them that are more trained in sensitivity to talk to parents or family members as it relates to a death of a loved one because they want answers, and dammit, we want answers too. But at that time, when I am distraught and you’re asking me, ‘Oh. Well, what time did you see him? Or what time did they leave?’ I mean, how can I give you that information if I am upset?” (SOV)
	“Have some compassion, man, for folks who have lost so many loved ones. You know, so of course, [LEOs] need to be trauma informed.” (HVIP representative)
	“Some people just don’t have that delicate touch…because a lot of times we are urgent…so we can get this perpetrator. But if you don’t come in, call, give [the patients] some time, let them gather their thoughts, you can bust it up.” (Detective)

Abbreviations: HVIP, hospital-based violence intervention program; LEO, law enforcement officer; SOV, survivor of violence.

**Table 3.** Quotations for Theme 3: Integrating Advocates for Survivors of Violence in the ED

Theme	Quotations from stakeholders
Need for support	“Yes, I think an advocate would be great because at a time like that, and truthfully, I don’t know the procedures for the program [and what is] restricted [in the] room. As a [survivor], the police can tell you anything whatsoever…some legal advocate will be great for anybody…someone that could potentially be there [in the ED], [to] let a [survivor] know their rights or just the procedure.” (SOV)
	“You want me to tell you something, and I am not even focused on what you’re asking me. I am more focused on [the fact that] my child is gone. So I think you need an advocate there, someone to talk for you when you can’t.” (SOV)
	“I used to ask the officer when I am getting ready to interview a patient, ‘Can you step out?’ And some do it, some don’t of course. But you know, just in my experience, they don’t have a lawyer present. They are in trauma. Patients are scared. And they are very vulnerable. So yeah, I am thinking [it] would really be a great thing [to have] lawyers.” (HVIP representative)
Holding law enforcement accountable	“A lawyer would have been helpful because he would know what to do. He wouldn’t allow for the police to take a statement without even me saying anything.” (SOV)
	“I think it needs to be some legal arm, maybe a lawyer. [LEOs] need to keep them body cams on too, because you never know…[law enforcement] needs more training, [SOVs] need an advocate, and [SOVs] need some legal person because a lot of times the police get out of line. We need an advocate and a legal person to make sure that everything’s done in a fine manner.” (SOV)

Abbreviations: ED, emergency department; HVIP, hospital-based violence intervention program; LEO, law enforcement officer; SOV, survivor of violence.

### Triaging Interactions With Law Enforcement

All stakeholder groups agreed that limiting or triaging interactions with law enforcement in the ED can assist in meeting different stakeholder priorities while promoting SOV recovery. In particular, most SOVs and HVIP representatives strongly advocated for limiting law enforcement interactions during SOV treatment and recovery, particularly in the acute phases of resuscitation. According to many SOVs, being interrogated while lacking capacity due to pain, medication, or trauma was a hindrance to their recovery, which is their main priority while in the hospital. For example, one patient stated, “Why is [law enforcement] even talking to me, because every word that I am saying…can be a minute detrimental to my life. I don’t think that was the time to have been asking me any questions.” Some SOVs highlighted that even when LEOs are “just doing their job,” the line and nature of questioning burden patients with managing the trauma of “what’s just occurred and then also what just occurred in terms of being in a hospital.”

Among SOVs, affected family members also recounted instances of law enforcement asking them to describe the traumatic incident soon after it occurred, deeming their timing to be inappropriate and emphasizing the need for space while grieving. One family member shared, “There were two detectives there [at the hospital], and they kept trying to ask me questions. And I am like, ‘My son is gone. I don’t know what to tell you.’” Another family member explained, “I think they should give family members their moment and their space. We are already hurting. You keep asking all these questions and it’s hard for us to be there.”

Notably, many LEOs agreed that formal hospital policies on receiving updates on patient conditions from hospitals could limit their time and presence in the ED. Law enforcement described that their main priority is to gather information for their investigations connected to the injury. As a result, timely updates on patient conditions, such as type of injury and health status, are important to maintaining the administrative flow of police investigations. Law enforcement recommended the asynchronous use of forms with items related to law enforcement priorities, separate private rooms in which LEOs can wait, or a hospital liaison for sharing updates to efficiently complete their duties while limiting direct patient contact or questioning. As a detective explained, “It could alleviate us having to come up to [the ED] if there was a person that could be a liaison for us.… We could maybe leave a detective’s name, who’s assigned to the case [and the liaison] could probably send them or the office an email update, maybe every 6 hours or something like that.”

HVIP representatives, who are violence prevention professionals, discussed their role in working with law enforcement to manage hospital interactions. These representatives described their duty to protect patients from “badgering” by law enforcement and effectively communicating updates with families to “try to reduce emotional distress.” Many HVIP representatives believed they could support law enforcement priorities and interactions with SOVs by serving as liaisons to offer trauma support to survivors. For example, an HVIP representative discussed how they can complement law enforcement goals while limiting SOV exposure to law enforcement: “We have been building connections with the police precincts just trying to help them understand that [HVIP staff] could be used as an alternative…and [SOVs] could come to us for trauma support that they really need.”

### Formalizing Hospital Staff and Law Enforcement Training

HVIP representatives and SOVs discussed the need for training hospital staff on their role in protecting SOV rights in the ED as well as the need for trauma-informed training for law enforcement. HVIP representatives agreed that hospitals need clearer policies and knowledge sharing on the roles and responsibilities of hospital staff in the ED. Specifically, they described a lack of understanding among hospital staff about what information they are obligated to share with law enforcement and the extent to which law enforcement is allowed access to SOVs. For example, an HVIP representative asked, “What do we have the right not to share with [law enforcement]? Because I think for a long time, and I think [LEOs] make you feel this way, that if [law enforcement] asks a question, you have to answer it.... What are the rights of [clinicians] to protect the health information of their patients? What is absolutely necessary that we tell the police? And can they limit communication with family?”

HVIP representatives also noted that law enforcement often restricts or prevents them from sharing important medical updates with family members in the ED. Without a clear understanding of their roles and responsibilities, many HVIP representatives reported being hesitant to enforce patient rights, such as asking law enforcement to leave the room or denying law enforcement access to patient belongings, thereby leading to unregulated law enforcement exposure for SOVs.

SOVs and HVIP staff emphasized the need for trauma-informed and sensitivity training for law enforcement when interacting with SOVs. Affected family members recounted several adverse experiences with law enforcement and underscored the gravity of sharing traumatic news of the death of a loved one. One family member stated, “I am not saying I don’t like [law enforcement], but I think they need sensitivity training…they don’t think about the parents that have just lost somebody.” Family members characterized the questioning by law enforcement at a time when family members are “distraught” as inappropriate. Additionally, some family members reported that law enforcement treated them like “criminals.”

Some LEOs also noted their lack of training on interacting in hospital settings, especially with SOVs. Without formal hospital training, law enforcement’s nature of interactions with SOVs can vary, sometimes being “badgering.” For example, a patrol officer stated, “I am sure you have seen it in your [ED], where [law enforcement] kind of oversteps the line, where perhaps we are even getting to the point where we are badgering, in your view, a wounded person. [A]s a [law enforcement] department, we should probably get some better training as to what our conduct should be in the hospital.”

### Integrating SOV Advocates in the ED

All stakeholder groups reached consensus on the benefits of integrating SOV advocates in the ED. SOVs and HVIP representatives shared the sentiment that advocates in the ED can relieve SOVs of the “full burden of self advocacy” and help protect their rights. Many SOVs noted that lawyers in the ED could help them understand their rights, the legal process after experiencing violence, and any support or benefits available to them as a survivor. SOVs also underscored the importance of legal support in holding law enforcement accountable. They discussed that having lawyers in the ED could prevent law enforcement from manipulating or coercing them and their families into providing information when they do not have the emotional and/or physical capacity to communicate clearly. For instance, one survivor verbalized their “right to recover”: “I think that the most important thing is that the first right should be given to a [survivor].… [T]hey have the right to a lawyer and have the right to remain silent for a recovery.… I feel like we should have the right to get medical attention. Without being affected by law enforcement.… Without being pressured by the police.”

Family members explained that having SOV advocates could help alleviate the burden of responding to immediate questions from law enforcement and communicate on their behalf when they are navigating trauma and grief. Law enforcement focus groups also had some general discussion highlighting the importance of having an advocate for SOVs who can serve as a “buffer” and focus on taking care of patients. Specifically, a detective described how sexual assault survivor advocates in the ED have supported law enforcement interactions with SOVs and prioritized SOV well-being: “[Sexual assault survivor advocates] make sure the [survivor] is comfortable and sometimes of course police, I guess, are intimidating, so [advocates] are the buffer.… That [advocate] is there for [the survivor’s] level of comfort and to help them through it, because [the advocate’s] role is totally just to take care of the [survivor] versus our role is to extract patient information so we can try to get the perpetrator.”

## Discussion

This study extended existing research on the adverse health, legal, and privacy ramifications of unregulated law enforcement presence in the ED. Specifically, it highlighted the importance of placing boundaries on law enforcement in the ED from a multistakeholder perspective. We found that triaging interactions with law enforcement, formalizing training of hospital staff and law enforcement, and integrating SOV advocates in the ED can help support diverse stakeholder priorities without compromising SOV rights and recovery. These strategies were agreed to be mutually beneficial by SOVs, HVIP representatives, and LEOs.

Our findings are responsive to the increasing demand for formal hospital policies and training on law enforcement interactions such as visitation, questioning, property collection, mandated reporting, and information sharing to help triage interactions in the ED.^5,12,20^ Most SOVs and HVIP representatives in this study advocated for triaging interactions with law enforcement in the ED, describing law enforcement interrogations as a hindrance to SOV recovery. Law enforcement in our study also supported triaging interactions to improve efficiency in meeting their investigative priorities. Prior research has documented the growing law enforcement presence in the ED, particularly in safety net hospitals, and law enforcement’s competing priorities in the ED.^1,2,3,5,12^ We identified points of collaboration between hospital and law enforcement stakeholders to limit interactions between SOV and law enforcement in the ED while supporting law enforcement in completing their mandatory duties. Key components of this shift include the potential use of preformulated, asynchronous information sharing to meet mandatory reporting obligations, protect privacy, and optimize efficiency. Additionally, our findings underscored that HVIP representatives are uniquely positioned to serve as liaisons for law enforcement in the hospital and may help limit unnecessary law enforcement presence in the ED. Many US jurisdictions also have mandatory reporting requirements, which can serve as guides on the exact information health care teams are obligated to share with law enforcement.^26^

While HVIP professionals are holistically trained to communicate with SOVs and provide trauma-informed support,^6,7,8,9,10^ our findings highlighted that HVIP representatives and LEOs also need formalized training on their roles and responsibilities in the ED. Most studies regarding law enforcement presence and hospital policies have focused on hospital staff and clinicians and shown a general lack of understanding of policies on law enforcement presence among these workers.^2,12,13,14,15^ The HVIP representatives we interviewed expressed similar themes, noting a lack of understanding regarding what information they must share with law enforcement and the limits of law enforcement in the ED. We extended this area of research to show that the lack of training was felt among LEOs as well. Officers in our study reported not receiving any formal training on how to conduct themselves in hospital settings and with SOVs, which can have severe public health implications. The lack of formalized training leaves room for unregulated and adverse interactions between SOVs and law enforcement, including perpetuating implicit biases,^12^ and should be addressed using trauma-informed and deescalation strategies.^27,28^ Recent efforts, such as the Active Bystandership for Law Enforcement project and Georgetown Law’s Center for Innovations in Community Safety, offer law enforcement training on trauma-informed principles to deescalate tension and optimize interactions between law enforcement, SOVs, and hospital stakeholders.^29^ Additionally, the HealthCARE Collaborative for Justice—an initiative by the Center for Innovations in Community Safety—has developed a set of hospital policies surrounding patient access, property, and use of restraints (alongside training on how to implement these policies) that can be used in and/or adapted to different hospital contexts.

Finally, all stakeholder groups had a consensus about the benefits of integrating SOV advocates in the ED. Previous studies have shown that advocates for SOVs (eg, sexual assault or rape survivor advocates) in emergency settings improve survivors’ experience and engagement with medical and legal systems.^30,31,32,33^ Our results corroborated the value of integrating SOV advocates in the ED to optimize hospital environments and prioritize SOV recovery. Depending on the structure and staffing of the individual HVIP, some of this advocate role may already be filled by HVIP staff. Our findings highlighted, however, that HVIP representatives lack formal training on managing law enforcement presence, which can impede their efforts for advocacy and supporting SOVs in the ED. In addition, legal protections are not widely established, including confidential and privileged communications between HVIP staff and SOVs as well as the right for HVIP staff to be present during law enforcement questioning and examinations.^10^ Some jurisdictions, however, have established such legal protections for sexual assault survivor advocates, which can be adapted for other SOV advocates.^34^

### Limitations

This study has some key limitations. First, it involved a single site, which may limit applicability to other settings. Nevertheless, the insights gained provide valuable guidance for designing trauma-informed interventions and increase support for SOVs in other EDs. Second, the mix of different data collection methods, formats, and periods to engage stakeholder groups may have introduced variation in responses, potentially complicating comparisons across groups. However, the flexible approach allowed the inclusion of SOVs, HVIP representatives, and LEOs—groups who are directly involved in the ED—in informing strategies that support SOV recovery in the ED. These findings fill a critical gap in the literature and offer insights into managing law enforcement presence in the ED. Finally, to support trust and minimize barriers to participation in this qualitative study, demographic questions were answered on an optional basis by participants. Consequently, demographic information was not collected in a consistent manner across stakeholder groups and is therefore not reported. Given that these data are not directly pertinent to the study aims, their absence does not constitute a key limitation. Future work should examine other hospital settings; include the perspectives of other stakeholders, such as nurses; and evaluate the implementation, large-scale feasibility, and impact of these identified strategies.

## Conclusions

To our knowledge, this work is among the first qualitative studies presenting multiple stakeholder perspectives on managing law enforcement presence in the ED. Our findings highlighted 3 main strategies, which emerged consistently across stakeholder groups, to manage law enforcement in the ED: triaging interactions with law enforcement, formalizing hospital staff and law enforcement training, and integrating SOV advocates in the ED. Optimizing hospital environments in emergency medical settings through formal policies and training on managing law enforcement presence in the ED has the potential to prioritize SOV recovery, limit interactions between SOV and law enforcement, and improve information sharing and job effectiveness for different stakeholders.

## Notes

Supplement.
Data Sharing Statement
