# Supplementary material for: Navigating Law Enforcement Presence in Emergency Departments
Source: JAMA Netw Open. 2026 Jan 13;9(1):e2551804. doi: 10.1001/jamanetworkopen.2025.51804 (PMC12801082; doi:10.1001/jamanetworkopen.2025.51804)
Supplement: Supplement. — Data Sharing Statement [file jamanetwopen-e2551804-s001.pdf]

Data Sharing Statement

Bhatnagar. Navigating Law Enforcement Presence in Emergency Departments. *JAMA Netw Open*. Published January 13, 2026. doi:10.1001/jamanetworkopen.2025.51804

Data
Data available: No
